# A Novel Multisegmental Individual-Based Exercise Approach for Postural Dysfunction and Balance Impairment in Older Adults: Randomized Controlled Trial

**DOI:** 10.2196/78426

**Published:** 2026-07-09

**Authors:** Filza Shoukat, Syed Shakil Ur Rehman

**Affiliations:** 1 Faculty of Rehabilitation and Allied Health Sciences, Riphah International University, 6 M Gulberg III Ferozepur Road Lahore, Lahore, 54600, Pakistan, 92 51591289

**Keywords:** exercise, old age, posture, balance, physical function

## Abstract

**Background:**

Age-related decline in mobility and balance substantially increases the risk of falls in older adults. Evidence-based exercise programs are recommended, but their effectiveness requires further evaluation.

**Objective:**

The study aimed to find out the effectiveness of multisegmental individual-based exercise program (MIBEX) approach for postural dysfunction and balance impairment in older adults.

**Methods:**

A 2-arm, single-blinded randomized controlled trial was conducted with 113 community-dwelling adults aged 65 years or older (exercise group, n=56; control group, n=57). Participants in the intervention arm received MIBEX, while the control group received postural education via video. Outcomes included the Timed Up and Go test, tragus-to-wall test, Mini-Balance Evaluation Systems Test, and postural sway, measured at baseline and 3 postintervention time points. Between-group comparisons were analyzed using the Mann-Whitney *U* test with effect sizes (*r*) and 95% CIs.

**Results:**

Significant between-group differences were observed across all outcomes (*P*<.01). The exercise group showed improvements in the Timed Up and Go test (median reduction from 15, IQR 13‐19 to 10, IQR 8‐12 s) compared with controls (15, IQR 13-18 to 12, IQR 10-14 s), with a large effect size (*r*=0.65, 95% CI 0.45‐0.80). Tragus-to-wall test performance improved markedly in the intervention group (median increase from 41, IQR 36-47 to 49, IQR 44-54 cm) versus controls (40, IQR 35-46 to 37, IQR 31-43 cm; *r*=0.52, 95% CI 0.30‐0.70). Mini-Balance Evaluation Systems Test scores declined in controls but improved in the intervention group (*r*=0.48, 95% CI 0.25‐0.66). Postural sway demonstrated moderate improvement (*r*=0.40, 95% CI 0.18‐0.58).

**Conclusions:**

These findings indicate that the MIBEX produced significantly greater improvements in mobility, balance, and postural stability compared with usual care, highlighting its potential as an effective intervention for older adults.

## Introduction

The 2019 United Nations World Population Prospects data show that approximately 8% of the global population is currently aged 65 years or older, and this figure is projected to reach almost 16% by 2050 [1]. The fastest-growing demographic population is the older adult population aged 80 years and above. Concerns are increasing regarding the increasing proportion of older individuals compared with the total population. It is estimated that by 2030, the percentage of older adults in the total population will increase by 9.3% [2]. According to the Global Burden of Disease Study, the percentage of years lived with disability has increased by 7.9% in men and by 6.5% in women from 1990‐2017 [3]. Approximately two-thirds of the older adult population lives in low- to middle-income countries, and like many others, Pakistan also experiences a demographic shift. This shift has significant health, social welfare, and economic development implications [4].

Aging is an ongoing and irreversible process resulting in changes in body composition, which affect physical fitness. Physical fitness decreases steadily with age [5]. Sarcopenia, the loss of muscle strength and mass due to biological aging, is a major cause of functional decline, dependency, and increased risk of falls and fall-related injuries in older adults [6]. Aging also results in various changes in the human body, including changes in body composition, such as increasing body fat, which is linked to a higher prevalence of chronic diseases such as diabetes, chronic lung disease, cancer, and stroke [6,7].

There is strong scientific evidence that regular physical activity provides extensive health benefits for people aged 65 years and above. In some cases, the evidence of health benefits is greater for older people because the negative outcomes associated with inactivity are more common in this age group than in younger individuals [8]. In 2020, the World Health Organization (WHO) updated its recommendations for sedentary behavior and physical activity for children and adolescents, adults, and older individuals.

According to these recommendations, an individual should engage in 150 to 300 minutes of moderate-intensity physical activity, 75 to 150 minutes of vigorous-intensity physical activity per week, or a combination of both [9]. Additionally, individuals are advised to participate in muscle-strengthening exercises targeting all major muscle groups at least twice a week. These recommendations highlight the importance of developing an effective program to promote physical activity for health-related purposes to reduce the risk of chronic illnesses, falls, injuries, morbidity, and premature mortality [10,11].

Different types of exercise programs have been used in previous studies to increase or maintain physical fitness [12] and body composition [13] in the older adult population. Multicomponent or multimodal exercise, which combines various exercise modalities, such as strength, balance, gait, aerobics, and flexibility, into a single exercise session, has gained increasing attention in recent years for improving the physical function of frail older adults [14-16].

Multicomponent physical exercise programs, particularly strength training, are recognized as the most effective approaches to delay the loss of strength [17,18] and functional disability, and to mitigate adverse effects such as hospitalization. Evidence-based systematic reviews and meta-analyses of randomized controlled trials have shown that multicomponent exercises have a positive impact on muscle mass, strength, balance, and physical performance [19-21]. The integration of multimodal exercise with functional balance and mobility training, moderate- to high-intensity progressive resistance training, and 30- to 45-minute sessions at least twice a week for optimal results is recommended [22].

An additional change that affects older adults, particularly females, is the alteration in postural control, which is influenced by hormonal changes, sarcopenia, osteopenia, and reduced physical activity. Postural imbalances contribute to an increased risk of falls [23]. Multicomponent exercises can help prevent falls by enhancing muscular function and postural control [24]. These exercises are recommended for the older population because they improve postural performance and increase muscle strength and power [25,26].

Although multicomponent exercise programs are known to improve physical function in older adults, most apply standardized protocols, and some studies address postural dysfunctions, such as thoracic kyphosis or forward head posture. Few interventions combine segment-specific training with structured postural education, and individualized progression based on functional assessment is rarely implemented. To address this gap, we developed a multisegmental individual-based exercise program (MIBEX), an equipment-free, cost-effective intervention that targets specific anatomical segments related to postural control, integrates postural correction strategies, and adjusts intensity and complexity weekly according to each participant’s needs. The objective was to assess its effects on balance, posture, and functional mobility in community-dwelling older adults. We hypothesized greater improvements in postural alignment and balance with this program compared to postural education alone.

## Methods

### Study Design

This was a 2-arm parallel design, single-blinded, single-center randomized controlled trial approved by the Ethical Committee of Riphah International University (RCRS-RE-PHD-RS/Fall19/127). The trial was registered at the Iranian Registry of Clinical Trials (trial ID IRCT20221005056094N1). The date of registration was October 25, 2022. All participants signed written informed consent before participating in the study. The participants were also informed that the study intervention had no disadvantages or risks. Careful monitoring of the participants was performed throughout the study, and necessary medical care was provided to the participants when needed. The participants were also informed that they could withdraw freely. During the entire study process, the identities of the participants were not disclosed in any publication of this study. The patient may choose not to participate in the study and withdraw their consent to participate at any time and will not be penalized in any way if they choose not to participate or withdraw from this study. The study was conducted according to CONSORT (Consolidated Standards of Reporting Trials) [27], and included the CONSORT checklist (Checklist 1).

### Recruitment and Follow-Up Periods

Participant recruitment and all study activities, including follow-up, were conducted between March 2022 and August 2022. The trial comprised a 12-week intervention phase immediately followed by a 12-week no-intervention follow-up period, with final assessments completed in August 2022. All primary and secondary outcomes, including those related to potential benefits and harms, were measured at baseline, during the intervention, post intervention, and at follow-up.

### Participants and Study Design

The data were collected at the outpatient department of the University Teaching Hospital, UOL, and in Lahore, Pakistan. A nonprobability convenience sampling technique was used to collect data from the geriatric population. The inclusion criteria were participants aged 65 to 75 years, including both male and female participants; those who could move independently and climb a flight of stairs independently; those who have thoracic kyphosis of 50 degrees or greater; and those who have cognitive function within normal limits, assessed by Mini-Mental State Examination ≥24. The exclusion criteria were participants who could not maintain balance when standing unassisted; those with diagnosed neurological or vestibular disorders; and those with serious medical conditions that would limit participation in the exercise program, that is, chest pain, myocardial infarction, or cardiac surgery within 6 months. Participants with uncontrolled hypertension and with a history of hip fracture or total hip replacement within the previous 12 months (due to its potential effect on balance and mobility outcomes) and those who used assistive devices for walking (eg, cane or walker) in daily activities were also excluded.

### Intervention

The intervention consisted of a 24-week exercise program. The treatment was applied twice weekly for 12 weeks, after which follow-up readings were taken for the next 12 weeks. Data from both the intervention group and the control group were obtained by the researcher at 6 different times: at baseline (T0) for 3 months (T1, T2, and T3), and then for the next 3 months (T4, T5, and T6) for follow-up visits. The details are shown in Table 1.

**Table 1.** Data collection and outcome measures scheduled per visit.

Activity/outcome measure	Baseline (wk 0)	Month 1	Month 2	Month 3	Month 4	Month 5	Month 6
Recruitment							
Participant selection	✓						
Allocation	✓						
Intervention							
Group A	✓	✓	✓	✓	✓	✓	✓
Group B	✓	✓	✓	✓	✓	✓	✓
Outcome assessment							
Postural sway	✓	✓	✓	✓	✓	✓	✓
Timed Up and Go test	✓	✓	✓	✓	✓	✓	✓
Mini-BESTest^a^	✓	✓	✓	✓	✓	✓	✓
Tragus-to-wall test	✓	✓	✓	✓	✓	✓	✓

a Mini-BESTest: Mini-Balance Evaluation Systems Test.

### Procedure

#### Overview

Baseline measurements of both groups were taken before treatment via a global postural system (GPS400). The GPS was used to measure postural sway as well as static and dynamic balance. Stabilometric assessment was performed via GPS by asking patients to stand on the platform while the participants stood on the postural analyzer for picture acquisition. The distance of the participant from the specific acquisition device was predetermined, and it was an essential requirement. Measurements were carried out on each acquired image, which was saved for future use. Postural sway was measured while the participants stood on a force platform for a 20-second period. This platform measured the displacement of the center of pressure (COP; sway path). Pictures were then taken first with the eyes open and then with the eyes closed. The participants were then asked to move on the platform to measure their dynamic balance. After the initial testing, the participants participated in 1-hour exercise sessions twice weekly for 12 weeks.

The exercise program was divided into 6 sections. Exercises were focused on improving balance, mobility, ﬂexibility, strength, and endurance, as well as providing postural education. To implement the program, the experimental group was supervised at all times by 2 physical therapists who were blinded to which older individuals were in each of the groups. The intensity and complexity of exercises were initially prescribed based on each participant’s baseline assessment. Adjustments were made weekly using the rating of perceived exertion (Borg scale) to ensure exercises remained challenging yet safe for all participants. The participants were divided into 2 groups.

#### Group A (Control Group)

Participants in the control group received postural education delivered twice weekly by a physiotherapist in small groups, with each session lasting 30‐45 minutes. The sessions used a postural correction video demonstrating proper sitting, standing, and walking posture, as well as exercises to improve body alignment during daily activities. Participants were encouraged to practice the demonstrated postures at home, and the sessions were reinforced with visual aids and *reminders* to ensure adherence. This component aimed to increase awareness of correct posture and support balance and functional performance, complementing the primary outcomes of the study. The postural education program administered to participants in the control group is described in Textbox 1.

Textbox 1. **Description of the control group.**

**Component**

- Postural education (control group)

**Description**

- Proper sitting, standing, and walking posture; exercises to improve body alignment

**Frequency and duration**

- Twice weekly; 30‐45 minutes per session

**Delivery method**

- Conducted by a trained physiotherapist in small groups of 6‐8; delivered via postural correction video

#### Group B (Exercise Group) MIBEX

The participants in this group received an exercise program. In accordance with the recommendations of the American College of Sports Medicine [28], every session consisted of the following exercises:

- Warm-up period (10 min)
- Exercise period (35 min)
- Postural education includes instructions for maintaining the correct posture (5 min)
- Cool-down period of stretching and relaxation exercises (10 min)

##### Warm-Up Period

It includes range-of-motion exercises for the shoulder, hip, knee, wrist, and ankle.

##### Exercise Period

It includes the following exercises:

###### Balance Exercises

Balance exercises for both static and dynamic balance were given to the patients. During the static exercises, the participants were instructed to remain in the same position for 10 seconds. After the samples were allowed to rest, the test was repeated 3 times. In the case of dynamic exercises, participants performed different exercises under different conditions that challenged their balance by demanding postural corrections. The exercises were also repeated 3 times.

###### Strengthening Exercises

Muscle-strengthening training consists of exercises for both the upper and lower limbs:

- Sets and repetitions: The participants performed from one set of 5 to 8 repetitions in the first training session to three sets of 10 to 12 repetitions at the end of the program.

- Intensity and progression: The intensity increased with increasing weight from 0.5 to 1.0 kg, and weight progression was determined at the individual level by the therapist.

###### Stretching and Flexibility Exercises

The upper limb (neck circles, shoulder circles, triceps stretch, pectoralis major stretch, and trapezius stretch) and the lower limb (quadriceps stretch, calf stretch, hamstring stretch, and back stretch) were used.

###### Endurance Exercises

These included brisk walking and running, starting first at a slow pace and then gradually increasing the intensity.

### Postural Education

Postural education included instructions for maintaining the correct posture (5 min).

### Cooling Down Period

The cool-down period consisted of stretching and relaxation exercises (10 min), and a progressive approach was used in all the multicomponent exercises.

A progressive approach was used in all the multicomponent exercises. On the basis of a gradual increase in training volume, sets, and repetitions, we intended to gradually include participants with a variety of different exercise levels.

A detailed description of the exercise program is given in Table 2.

**Table 2.** Detailed description of the multisegmental individual-based exercise program.

Exercise protocol	Description of exercises	Duration (min)	Intensity or progression
Warm-up period	ROM^a^ exercises for hip, knee, ankle, shoulder, and wrist	10	Continue as such for 24 wk
Stretching exercises	Upper limb: Neck circle, shoulder circle, triceps stretch, pectoralis major stretch, trapezius stretch Lower limb: Quadriceps stretch, calf stretch, hamstring stretch, and back stretch	10	Week 1‐4: 5 min Week 5‐8: Continue above Week 9‐12: 10 min Week 13‐24: Continue above
Strengthening exercises	Bicep curl, triceps curl, hip extension, hip flexion, hip abduction, and knee extension	10	Week 1‐4: 8 reps×2 sets, intensity 65% of 1 RM^b^ Week 5‐8: 10 reps×3 sets, intensity 75% of 1 RM Week 9‐12: 12 reps×3 sets, intensity 85%‐90% of 1 RM Week 13‐24: Maintain above
Endurance exercises	Brisk walk, running, and jogging (individualized; low-impact alternatives such as slow-paced walking if needed)	10	Week 1‐4: 10 min at comfortable speed Week 5‐8: 5 min warm-up, then increase speed for remaining duration Week 9‐12: 10 min at increased speed Week 13‐16: 15 min at maximum tolerated speed or intensity
Balance exercises	Heel raises, 1-leg stand, toe raises, sit-to-stand, marching (hold each for 10 s)	10	Week 1‐4: 2-hand support Week 5‐8: 2-hand support Week 9‐12: 1-hand support Week 13‐24: No support
Postural education	—^c^	5	Twice weekly
Cool-down period	Stretching and relaxation exercises	5	Continue as such for 24 wk

a ROM: range of motion.

b RM: repetition maximum.

c Not applicable.

### Outcome Measures

Four primary outcome measures were used to evaluate the effects of the MBIEX intervention.

#### Postural Sway

Postural sway refers to the continuous, small movements of the body’s COP during quiet standing, reflecting postural stability and balance control. In this study, postural sway was assessed using the GPS**,** which measures the quantitative measure of overall body sway [29]. Measurements were conducted under eyes-open and eyes-closed conditions while participants stood in a comfortable upright position. Lower sway values indicate greater postural stability and better balance control. The GPS system provides reliable and objective measurements, enabling accurate assessment of subtle changes in postural control [30]. Recent studies have reported good test-retest reliability for COP measures obtained via force platforms (intraclass correlation coefficient, ICC=0.85‐0.95), supporting the use of GPS in clinical and research settings. Postural sway is widely used as a sensitive outcome to evaluate the effectiveness of interventions aimed at improving balance and reducing fall risk in older adults [31].

#### Timed Up and Go Test

This test was developed by Podsidalo and Richardson [32]. The functional mobility of independent people was evaluated via the Timed Up and Go (TUG) test. A stopwatch was used to record the time it took to stand from a chair, walk a 3-meter distance at the usual speed, turn around, and sit down again. Timing began when the instructor gave the command to go and ended when the participant returned to the starting position. The TUG test has excellent test-retest reliability (ICC=0.97) [33]. A TUG performance of less than 20 seconds indicates mobility independence, whereas a performance of more than 30 s indicates dependence [34].

#### Mini-Balance Evaluation Systems Test

The biomechanical constraints, stability limits or verticality, anticipatory postural adjustments, postural responses, sensory orientation, and stability in gait subsystems of the 6 subsystems that underlie static and dynamic balance are covered by the 14 items of the Mini-Balance Evaluation Systems Test (Mini-BESTest), a clinical assessment tool for measuring balance. The Mini-BESTest’s test-retest reliability for people with a range of neurological conditions and balance issues has also been demonstrated to be outstanding (ICC=0.92-0.97) [35]. The maximum score on the 14-item Mini-BESTest, which is graded from 0 to −2, is 28. Only the lower scores for items 3 (single-leg stance) and 6 (compensatory lateral stepping) were used when the overall score was calculated. For item 14, the score should be reduced if a person’s gait slows by more than 10% between the TUG with and without a dual task [36].

#### Tragus-to-Wall Test

It is a reliable test of forward-flexed posture in healthy younger (20‐30 y) and older (>60 y) adults. The patient stood with their feet separated by the length of the individual’s foot; heels 10.0 cm from the wall, and buttocks and posterior thorax against the wall; head looking straight ahead: level being horizontal between the bottom of the nose and the tragus. The distance between the posterior aspect of the tragus and the wall was measured with a ruler [37].

### Sample Size

The study sample size was estimated on the basis of the mean and SD reported by Szturm et al [38] for the balance scale. An open-source online calculator (OpenEpi V.3.00) was then used to estimate the total sample size as 91 patients. To account for potential attrition, an anticipated dropout rate of 20% was incorporated, consistent with rates reported in previous longitudinal exercise intervention studies involving older adults [39]. This adjustment was made to minimize the risk of reduced statistical power due to participant loss from health-related issues, personal circumstances, or loss to follow-up. Accordingly, the final estimated sample size was 113 patients, with 56 allocated to the experimental group and 57 to the control group. A 95% CI (α) and 80% power were used in the calculation.

### Randomization and Blinding

Participants were recruited using convenience sampling and randomly assigned to 1 of 2 groups using the simple lottery method. Each participant was given a unique identification number by the principal investigator, which was written on identical slips of paper and sealed in opaque envelopes. These envelopes were thoroughly mixed and placed in a box. A research assistant—independent of the recruitment, treatment, and assessment process—randomly drew envelopes to assign participants to either group, thereby ensuring allocation concealment and maintaining a 1:1 ratio between groups.

Due to the nature of the interventions, blinding of the participants and treating therapists was not feasible. However, to minimize detection bias, the outcome assessor remained blinded to group allocation throughout the study. Additionally, the data provided to the statistician were anonymized and coded (eg, as group A and group B) to ensure blinding during analysis.

### Statistical Analysis

All statistical analyses were performed using SPSS version 26 (IBM Corp.). Continuous variables such as age, weight, and height were reported as mean (SD). The normality of the data was checked with the Kolmogorov-Smirnov test. For comparisons between groups at each time point (baseline, 3 mo), we used the Mann-Whitney *U* test. For within-group changes over time (eg, baseline vs 3 mo), we applied the Wilcoxon Signed-Rank Test. When more than 2 time points were compared within a group, we first ran the Friedman test, followed by pairwise Wilcoxon tests with Bonferroni correction to adjust for multiple comparisons. In addition to *P* values, effect sizes (*r*) were calculated for all nonparametric tests using the formula *r=Z*/√*N* (n=number of observations in the test), along with 95% CIs for *r* to provide an estimate of the magnitude and precision of effects. Statistical significance was set at *P*<.05.

### Ethical Considerations

The study was approved by the Ethical Committee of Riphah International University (RCRS-RE-PHD-RS/Fall19/127). All participants signed written informed consent before participating in the study. The subjects were also informed that the study intervention had no disadvantages or risks. Careful monitoring of the participants was performed throughout the study; necessary medical care was also provided to the participants when needed. During the entire study process, the identities of the participants will not be disclosed in any publication of this study. The patient may choose not to participate in the study and withdraw their consent to participate at any time and will not be penalized in any way if they choose not to participate or withdraw from this study. All the participants participated voluntarily in the study.

Completed personal data or other documents containing protected personal health information were kept in a locked file at the principal investigator’s office. The data were entered into an electronic deidentified database by authorized study team members and checked for completeness and accuracy. Access to data with identifiers was restricted to authorized study team members and authorities. The electronic data were kept on a server regulated by the local research institute. Data will be destroyed 10 years after the study’s finalization or 5 years after the last publication.

## Results

### Participant Flow and Baseline Characteristics

**Figure 1.**
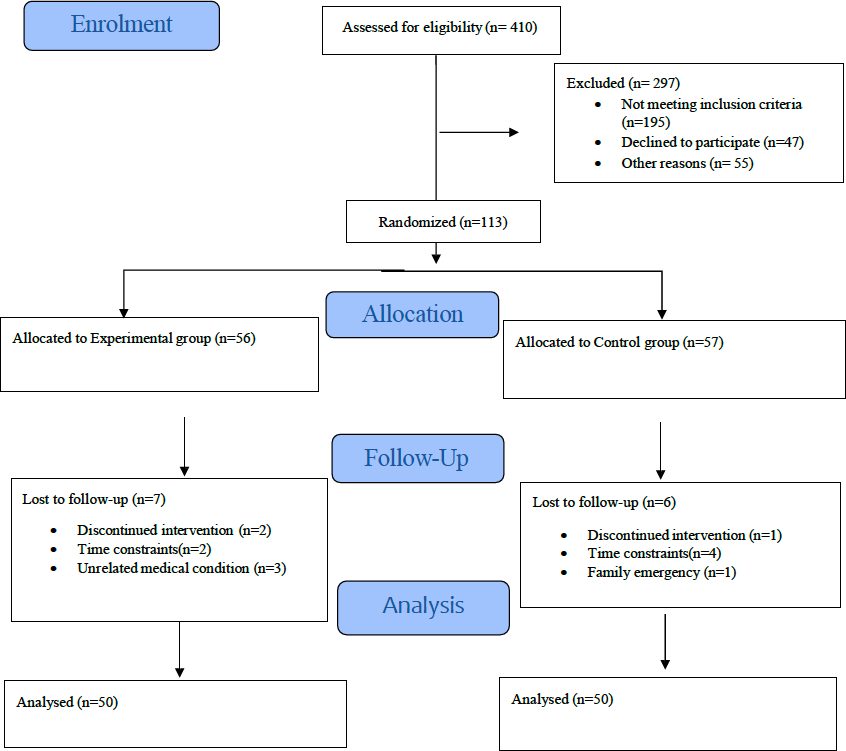
CONSORT (Consolidated Standards of Reporting Trials) flow diagram.

Figure 1 provides a detailed overview of participant enrollment, allocation, follow-up, and analysis. A screening of 410 healthy older individuals was performed for participation in the study. After the exclusion of 297 participants, 113 fulfilled the inclusion criteria of the study. Fifty-seven participants were allocated to the control group, and 56 were allocated to the exercise group. During the study, 13 participants dropped out, and 100 participants completed the study for 24 weeks. The overall schematic flow of the patient sample is given in the CONSORT guidelines. There were no statistically significant differences between the groups in terms of demographic or clinical characteristics at baseline.

### Basic Demographics

Descriptive statistics of gender, age, sex, weight, height, and BMI were included as the means and SDs and are summarized in Table 3.

Table 3 shows that the groups were comparable in terms of gender distribution, age, height, weight, and kyphosis angle, with no statistically significant differences observed (*P*>.05). A significant difference was noted for BMI, which was slightly higher in the exercise group compared to the control group (*P*=.04).

**Table 3.** Baseline demographics of study participants.

Variables	Control group	Exercise group	*P* value
Gender, n (%)			
Men	11 (22)	12 (24)	.81
Women	39 (78)	38 (76)	.42
Age (y), mean (SD)	70.36 (3.08)	70.34 (2.99)	.97
Height (cm), mean (SD)	181.0 (14.0)	178.0 (15.0)	.29
Weight (kg), mean (SD)	71.52 (5.36)	72.34 (6.01)	.47
BMI (kg/m²), mean (SD)	25.42 (1.30)	25.99 (1.38)	.04
Kyphosis (°), mean (SD)	51.90 (4.83)	50.78 (5.20)	.27

Table 4 summarizes the between-group comparisons of all primary outcome measures using the Mann-Whitney *U* test. The exercise group demonstrated significantly greater improvements than the control group in TUG (*U*=161.00; *P*<.001; *r*=0.65, 95% CI 0.45‐0.80), tragus-to-wall test (TWT; *U*=654.00; *P*<.001; *r*=0.52, 95% CI 0.30‐0.70), Mini-BESTest (*U*=560.00; *P*<.001; *r*=0.48, 95% CI 0.25‐0.66), and postural sway (*U*=480.00; *P*=.002; *r*=0.40, 95% CI 0.18‐0.58). The magnitude of effect sizes suggested a moderate-to-large effect of the intervention on mobility and balance outcomes.

**Table 4.** Comparison of outcome measures between the groups.

Outcome measure and groups	Baseline, median (IQR)	Postintervention 1, median (IQR)	Postintervention 2, median (IQR)	Postintervention 3, median (IQR)	Mann-Whitney *U*	*P* value	Effect size, *r* (95% CI)
TUG^a^ test (s)							
Control	15 (13‐18)	14 (12‐16)	13 (11‐15)	12 (10‐14)	161.000	<.001	0.65 (0.45‐0.80)
Exercise	15 (13‐19)	12 (10‐14)	11 (9‐13)	10 (8‐12)	—^b^	—	—
TWT^c^ test (s)							
Control	40 (35‐46)	39 (34‐45)	38 (32‐44)	37 (31‐43)	654.000	<.001	0.52 (0.30‐0.70)
Exercise	41 (36‐47)	45 (40‐50)	45 (40‐50)	49 (44‐54)	—	—	—
Mini-BESTest^d^ (score)							
Control	21 (19‐25)	20 (18‐23)	19 (17‐22)	18 (16‐21)	560.000	<.001	0.48 (0.25‐0.66)
Exercise	20 (18‐24)	24 (22‐28)	26 (23‐29)	27 (24‐30)	—	—	—
Postural sway (cm)							
Control	3.2 (2.8‐3.7)	3.0 (2.6‐3.5)	2.9 (2.5‐3.4)	2.8 (2.4‐3.3)	480.000	.002	0.40 (0.18‐0.58)
Exercise	3.3 (2.9‐3.8)	2.6 (2.2‐3.0)	2.4 (2.0‐2.8)	2.2 (1.8‐2.6)	—	.002	0.20 (0.16‐0.38)

a TUG: Timed Up and Go.

b Not applicable.

c TWT: tragus-to-wall test.

d Mini-BESTest: Mini-Balance Evaluation Systems Test.

Table 5 presents the within-group changes in functional performance and postural control outcomes for the exercise and control groups across baseline, month 1, month 2, and month 3 assessments. Both groups demonstrated statistically significant improvements in TUG, TWT, Mini-BESTest, and postural sway (*P*<.001 for most outcomes), with the exercise group showing larger effect sizes (*r*=0.71‐0.80) compared to the control group (*r*=0.40‐0.70). The exercise group exhibited the greatest gains in mobility and balance measures. Table 5 shows between-group comparisons of all primary outcome measures using the Mann-Whitney *U* test. At postintervention 3, the exercise group performed significantly better than the control group on the TUG test (median 10, IQR 8-12 vs 12, IQR 10-14 seconds; U=161.00, *P*<.001; *r*=0.65, 95% CI 0.45-0.80), TWT (49, IQR 44-54 vs 37, IQR 31-43 cm; U=654.00, *P*<.001; *r*=0.52, 95% CI 0.30-0.70), Mini-BESTest (27, IQR 24-30 vs 18, IQR 16-21 points; U=560.00, *P*<.001; *r*=0.48, 95% CI 0.25-0.66), and postural sway (2.2, IQR 1.8-2.6 vs 2.8, IQR 2.4-3.3 cm; U=480.00, *P*=.002; *r*=0.40, 95% CI 0.18-0.58). These findings suggest moderate-to-large intervention effects for mobility and balance outcomes. These results indicated that the MIBEX effectively enhanced postural stability and functional mobility over the intervention period.

**Table 5.** Comparison of outcome measures within the group.

Outcome measure and groups	Baseline, median (IQR)	Month 1, median (IQR)	Month 2, median (IQR)	Month 3, median (IQR)	*P* value	Wilcoxon *z*-score	Effect size (*r*)	95% CI for change
Timed Up and Go test (s)								
Control	9.5 (8.6‐10.3)	6.8 (6.2‐7.4)	5.1 (4.6‐5.6)	4.8 (4.3‐5.3)	<.001	−4.45	0.70 (large)	0.52‐0.84
Exercise	17.3 (16.4‐18.3)	15.5 (14.5‐16.4)	13.9 (13.0‐14.9)	12.2 (12.0-11.4)	<.001	−5.12	0.80 (large)	0.65‐0.90
Tragus-to-wall (s)								
Control	3.7 (3.3‐4.2)	6.6 (5.9‐7.3)	9.2 (8.7‐9.7)	10.4 (9.9‐10.9)	<.001	−4.38	0.66 (large)	0.47‐0.81
Exercise	9.8 (9.2‐10.4)	7.4 (6.9‐7.9)	8.2 (7.7‐8.7)	6.1 (5.6‐6.5)	<.001	−4.71	0.71 (large)	0.55‐0.85
Mini-BESTest^a^ (score)								
Control	3.7 (3.3‐4.2)	6.6 (5.9‐7.3)	9.2 (8.8‐9.6)	10.4 (9.6‐10.2)	<.001	−3.98	0.60 (large)	0.40‐0.77
Exercise	4.0 (3.5‐4.5)	3.7 (3.2‐4.1)	7.9 (7.6‐8.2)	8.4 (7.1‐8.3)	<.001	−4.89	0.77 (large)	0.63‐0.89
Postural sway (cm)								
Control	3.4 (3.0‐3.8)	2.9 (2.5‐3.3)	2.6 (2.2‐3.0)	2.5 (2.1‐2.9)	<.001	−4.32	0.65 (large)	0.45‐0.80
Exercise	4.8 (4.4‐5.2)	3.8 (3.4‐4.3)	3.5 (3.1‐4.0)	—^b^	<.001	−4.78	0.72 (large)	0.55‐0.85

a Mini-BESTest: Mini-Balance Evaluation Systems Test.

b Not applicable.

### Adherence to the Intervention

Adherence to the intervention was tracked using attendance logs maintained by the treating physiotherapist. Participants were encouraged to attend at least 75% of scheduled supervised sessions over the 12-week intervention period, followed by a 12-week follow-up (no adherence data were collected during the follow-up). Adherence rates for both groups during the 12-week intervention period are presented in Table 6. The intervention group maintained high attendance (92% at wk 4% and 87% at wk 12), whereas the control group’s adherence declined from 78% to 70% over the same period.

**Table 6.** Adherence rates for the intervention and control groups over the 12-week supervised intervention period.

Time point	Intervention group (%)	Control group (%)
T1 (wk 4)	92	78
T2 (wk 8)	89	74
T3 (wk 12)	87	70

### Adverse Events

Adverse events were monitored throughout the intervention period by the treating physiotherapist and during each exercise session. Participants were instructed to promptly report any discomfort, injury, or health-related incidents at any time during the study. No adverse events were reported by participants in either the experimental group or the control group during the study period.

## Discussion

### Principal Findings

This study focused on assessing the impact of a multisystemic exercise program on the physical function of community-dwelling older adults. This study revealed that a multisystemic exercise program, including low-to-moderate intensity resistance, cardiorespiratory, balance, strength, and flexibility training, along with postural education and an increase in moderate-to-vigorous intensity exercise performed twice a week for 24 weeks, significantly improved physical function and reversed the decline in function associated with aging. These findings support the original hypothesis that an exercise intervention positively and substantially affects physical fitness in community-dwelling older adults.

The participants in this community-based exercise program achieved considerable gains in multiple functional capacities with minimal investment in equipment compared with conventional strength and/or cardiorespiratory training using costly devices [6]. The improvements were similar to those reported in a recent review of 28 studies on resistance training [40]. Consequently, multicomponent exercise programs may be beneficial, as there is a clear connection between high physical activity and low sedentary behavior, as well as improved muscle strength in physically active older adults [41]. Meta-analyses of recent research have shown that multicomponent exercise programs effectively enhance various aspects of health-related physical fitness [14,38].

The multicomponent exercise program significantly improved lower and upper limb strength. This improvement is likely due to the stimulus provided by the exercise program and the adherence to the exercise prescription guidelines [42]. As participants perform strength exercises, they place demands on their muscles, which stimulates muscle fibers and leads to an increase in muscle strength. Progression training increased over the 24 weeks during which participants performed more upper limb and lower limb exercises. These findings align with those of the Beck Jepsen et al study [43], and they support the results of a recent meta-analysis that emphasized the importance of multiple sets in producing benefits in muscle strength [44].

A systematic review of randomized clinical trials with frail older patients revealed conflicting data regarding improvements in the risk of falls, mobility, balance, strength, functional capacity, and body composition [45]. Even among those who showed improvements in functional capacity, attributing the success of these results to a specific element of the intervention protocol is not appropriate.

In addition, multiple systematic reviews have shown that the multicomponent training regimen, comprising sessions that last 30 to 45 minutes and are conducted 3 times per week, is the most commonly recommended protocol for exercise among frail older adults [46]. Specifically, multicomponent exercise programs that incorporate resistance, balance, and flexibility exercises are highly effective for this population [41,42]. Our study is consistent with these findings, as we used 60-minute exercise sessions twice weekly for a 24-week duration. A year ago, a study in which a TWT was used to measure the altered angle was conducted to investigate the effects of exercise programs on age-related kyphosis. A comparison of the preintervention and postintervention values of the occiput-to-wall test revealed a significant difference after the protocol was administered (*P*<.001) [43], similar to our study’s results. Multicomponent exercise programs delivered in primary health care settings have been shown to improve postural control and muscle power in older adults, potentially helping to prevent falls and enhance quality of life, as demonstrated in a study with a similar design to ours conducted on healthy older individuals. The sample size of that study was small, with participants averaging 60 years of age [24].

The main strength of our study is that it uses a multicomponent exercise program (MIBEX), which appears to be a feasible, efficient, and cost-effective option for promoting physical activity among older adults in primary health care settings. This is because the exercises can be performed in groups, require inexpensive equipment, and can be modified to suit local characteristics. Additionally, our study uses a personalized exercise program tailored to individual characteristics and includes postural education.

This study had several limitations. First, it was not possible to blind participants, clinicians, or trainers in this type of exercise program. Although the outcome assessors were blinded to the groupings, some participants may have inadvertently revealed their treatment status, which could have affected the results. This study used a postural teaching program via video for the control group. However, there was no significant difference in the outcome measures since participants did not adhere to the activity, leading to a low rate of participation in the exercise sessions. Despite frequent stimulation and customized work, program adherence fell short of expectations.

Moreover, participation was entirely voluntary, and the main cause for exclusion was when participants refused to participate. As a result, the majority of participants may have established a positive relationship between exercise and health, which could have led to bias. Additionally, we could not determine whether participants who declined to participate would have benefited from physical activity as well.

Future studies should specifically consider the effects of exercise programs on physical performance by gender. Evidence suggests that older women may experience greater declines in physical function and show less pronounced improvements in strength and balance following exercise interventions compared to men [47]. Therefore, future research should account for sex as an important factor when designing and evaluating exercise programs, and may consider including balanced numbers of male and female participants to better assess gender-specific responses.

The current findings can be used to plan and implement future useful public health interventions for community-dwelling older individuals, which could reduce prospective healthcare expenses associated with disabilities among this population. Additionally, primary health care programs should aim to minimize barriers to physical activity promotion. Although recommendations from the literature should be considered in exercise programs for older adults, they may occasionally create barriers to the development of interventions. Nonetheless, socialization and recreational activities seem to be essential components in enhancing adherence among older adults and should be incorporated into multicomponent exercise programs.

### Conclusion

The 24-week tailored exercise program had significant positive effects on improving physical function in older adults. The participants exhibited marked improvements in strength, flexibility, balance, and endurance, leading to better daily performance, increased mobility, and a reduced risk of falls. The personalized nature of the program ensured higher adherence rates and more meaningful outcomes, addressing various aspects of physical function for overall health and well-being.

## Supplementary material

10.2196/78426 Checklist 1 CONSORT checklist.

## Abbreviations

CONSORT: Consolidated Standards of Reporting Trials
COP: center of pressure
GPS: global postural system
ICC: intraclass correlation coefficient
MIBEX: multisegmental individual-based exercise program
Mini-BESTest: Mini-Balance Evaluation Systems Test
TUG: Timed Up and Go
TWT: tragus-to-wall test
WHO: World Health Organization
